# On Exact Outage and Throughput Performance of Cognitive Radio based Non-Orthogonal Multiple Access Networks With and Without D2D Link

**DOI:** 10.3390/s19153314

**Published:** 2019-07-28

**Authors:** Dinh-Thuan Do, Anh-Tu Le, Chi-Bao Le, Byung Moo Lee

**Affiliations:** 1 Wireless Communications Research Group, Faculty of Electrical and Electronics Engineering, Ton Duc Thang University, Ho Chi Minh City 700000, Vietnam; 2 Faculty of Electronics Technology, Industrial University of Ho Chi Minh City (IUH), Ho Chi Minh City 700000, Vietnam; 3 School of Intelligent Mechatronics Engineering, Sejong University, Seoul 05006, Korea

**Keywords:** non-orthogonal multiple access (NOMA), cognitive radio, device-to-device, outage probability

## Abstract

In this paper, we investigate the performance of a secondary network in a cognitive radio network employing a non-orthogonal multiple access (NOMA) scheme to form a CR-NOMA system serving many destination users. In the secondary network of our proposed system, a device-to-device (D2D) scheme is deployed to further provide the signal transmission at a close distance of NOMA users in downlink, and such performance is evaluated under the situation of interference reception from the primary network. An outage performance gap exists among these NOMA users since different power allocation factors are assigned to the different destinations. Unlike existing NOMA schemes that consider fixed power allocation factors, which are not optimal in terms of outage performance, our proposed paradigm exhibits optimal outage in the scenario of D2D transmission. In particular, the outage performances in two kinds of schemes in term of existence of D2D link are further achieved. Simulation results validate the analytical expressions, and show the advantage of each scheme in the proposed CR-NOMA system based on outage performance and throughput.

## 1. Introduction

As a spectrum sharing model, cognitive radio (CR) can enhance traditional statistical spectrum utilization by permitting a secondary user (SU) to access the resource of the primary user (PU) when there is idle spectrum. There are two popular kinds of novel technologies to satisfy the demands of spectrum resources, namely overlay CR and underlay CR. The first one dynamically chooses vacant channels and does not have inter-user interference, while the second one chooses channels which have a certain interference temperature. As a main condition, the SU is constrained to make a harmless impact on the normal communications in the network containing the PU. By employing spectrum sensing, the SU senses the idle spectrum [1,2,3]. In the traditional schemes, when the absence of the PU is recognized, the SU can only access to the idle channel. Moreover, the SU is required to leave the occupied channel if there exists the presence of the PU. There is a widely deployed energy detection strategy to detect the PU by comparing a determined threshold to the accumulated energy statistics of the PU signal. The PU is known to be absent in the case that any energy statistics are less than their thresholds [4,5,6,7].

To implement the future wireless networks, Non-Orthogonal Multiple Access (NOMA) is proposed as one of the strong candidates since it supports massive connectivity and high spectral efficiency [8]. To achieve massive users on the same radio resources block (i.e., frequency and time), NOMA users are simultaneously served by splitting them into power domain. To address such ability, superposition coding (SC) is implemented at the transmitter and successive interference canceler (SIC) is required at the receivers. Power domain based NOMA was firstly recommended for the future radio networks in [9]. NOMA benefits from its superiority compared to the orthogonal multiple access (OMA) techniques in terms of the overall system capacity. The physical layer security was implemented to enable secure transmission in the context of NOMA, and the secure outage probability of downlink NOMA system was studied in [10], and it is further recommended to deploy in the Internet of Things (IoT) system. Extended coverage and reliable transmission are benefited from various schemes of cooperative relaying network reported in [11,12,13,14,15,16,17,18]. The authors of [19,20,21] introduced a cooperative relaying network using the architecture of NOMA. Device-to-device (D2D) transmission was proposed to deploy NOMA and the outage behavior performance was evaluated [19]. The applications of NOMA with the other relaying techniques also have received a great deal of attention to exhibit the reliability of such cooperative NOMA communication, which is considered one of the most investigated topics. The worse case of NOMA under a situation of imperfect channel state information (CSI) was investigated in [20], and the achievable outage probability was analyzed. In addition, other cooperative-NOMA was examined in [21,22,23] based on the assumption that the strong users have prior knowledge of the weaker users’ messages. In these schemes, relay can act in a role of the user who intends to serve a far user in a downlink NOMA scheme. The outage probability and the diversity order of the cooperative-NOMA were analyzed in [21,22] with the wireless power transfer technique. In these NOMA networks, a single antenna node was assumed. In [23], the authors extended the analysis in [21,22] and assumed the node is equipped with multiple antennas.

The combined architecture of NOMA and CR, namely CR-NOMA, was designed in a cooperative NOMA [24,25,26,27]. As a promising technique, an underlay cognitive radio network (CRN) with NOMA was proposed to solve the problem of scarce spectrum. To improve the transmission secrecy, such novel NOMA-enabled underlay CRN can be employed for the deliberately introduced interference. For example, NOMA with imperfect successive interference cancellation (SIC) was explored in an underlay CR network [24]. To evaluate CR-NOMA under a situation when eavesdroppers overhear a legal signal, secure analysis is an open problem in such CR-NOMA. In [25], a NOMA system was investigated in terms of secure performance. Considering on degraded performance due to the imperfect CSI, the authors indicated optimal power allocation coefficients for different distances of the users to achieve the outage probability fairness for both users, and the proposed system model showed the superiority of the CR-NOMA compared to cooperative orthogonal multiple access (OMA) [27].

To meet both the high-throughput requirement and massive connectivity, the authors of [28,29,30,31,32,33] explored Device-to-device (D2D) communications. For example, D2D-enabled dense heterogeneous networks (HetNets) with NOMA was studied in [28]. In particular, they considered joint power allocation and user scheduling to maximize the ergodic sum rate of the near users (NUs) located in the small cells while guaranteeing the quality-of-service requirements of the far user [28]. They confirmed that the NOMA technique invoked to serve more downlink users simultaneously. D2D-enabled multi-hop transmission was deployed to improve signal reception of the far users on the cell edge. The authors of [29] introduced D2D communications which are containing an uplink cellular system and sparse code multiple access (SCMA) technology. In such SCMA-assisted D2D network, the base station (BS) can decode the signals of cellular users without mutual interference. Mobile edge computing (MEC) and NOMA have been proposed as the promising techniques in [29]. The authors of [30] considered how to minimize the weighted sum of the energy consumption and delay in all users by jointly optimizing the channel allocations, computing resource, and power. More specifically, they recommended a novel power allocation algorithm using the particle swarm optimization (PSO) to apply in the single NOMA group including multiple cellular users. In [31], the resource allocation problem for D2D underlaid cellular networks was examined in terms of the uplink multi-carrier NOMA.

Motivated by recent works [27,31], this paper considers a new cooperative CR-NOMA system using with and without a D2D link, where the secondary network with the fixed power source was evaluated to satisfy the interference constraint for the primary network. In recent work [27], they did not consider situation as two NOMA users located in proximity scenario. In such circumstance, D2D is an efficient way for two users to communicate with high speed and high capacity. This is a challenging situation, as the primary network in the considered CR-NOMA degrades the performance of the secondary network with existence of interference and other transmit power constraints. These analyses are main motivation for this paper, which examines a new system model that merges schemes including NOMA and D2D into CR-NOMA. Such a NOMA scheme is designed to help improve the signal quality by forwarding the signal to a representative weak user from a strong user in NOMA.

The main contributions of this work are summarized as follows.

- We examine a new D2D transmission using a system model developed in [27]. Different from the results in [27], in this paper, D2D supports a new transmission for close users who are located in a narrow cluster in a normal cellular network.
- We formulate two cases of analytic expressions that consider existence/non-existence of a D2D link in the CR-NOMA of secondary network under interference constraint from the primary network. The underlay CR-NOMA provides an ability to serve D2D transmission. We show that two NOMA users served by the BS exhibit a performance gap in terms of outage behavior.
- The formulated problem of outage probability is difficult to evaluate, and thus, to tackle this challenge, an asymptotic analysis is provided to present more insights into the proposed system.
- Extensive simulation results are provided and the simulation results show that there is a trade-off between the transmission SNR at the BS of a secondary network and the outage behavior of each user in the context of NOMA. Besides, the interference term originated from a primary network also impacts the performance of UEs in the secondary network.

The rest of this paper is organized as follows. In Section 2, the system model is presented. Section 3 presents the formulated problem in term of outage behavior and its asymptotic analysis. Section 4 presents a more challenging scenario to provide D2D transmission along with their outage performance. Section 5 presents numerical results. Finally, the paper is concluded in Section 6.

## 2. System Model

**Figure 1:**
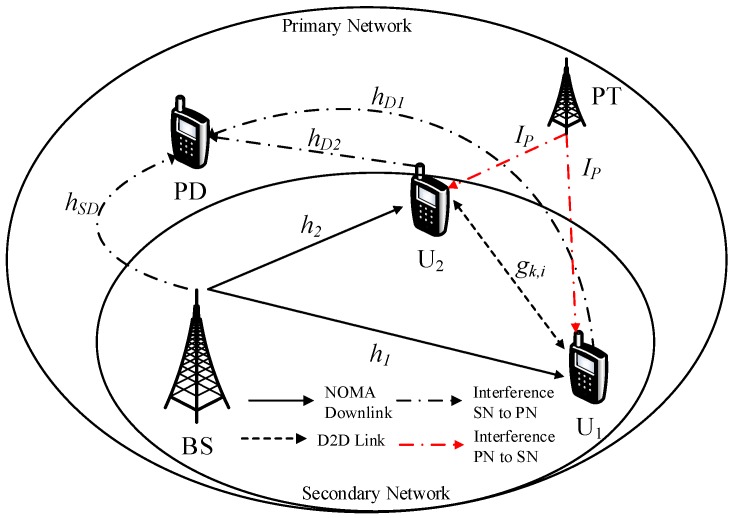
System model of CR-NOMA employing D2D link.

The system model is shown in Figure 1 and such a system comprises primary network (PN) and underlay secondary network (SN). In this model, there are two transmit sources, called base stations (PT and BS in Figure 1), and three users. PD is the user in the primary network while $U_{1},U_{2}$ are two users in the secondary network. A relaying scheme is employed at SN to perform transmission from a base station (BS) to two device-to-device (D2D) users ($U_{1},U_{2}$) and D2D transmission is supported by those two users. It is worth pointing out $U_{1},U_{2}$ are considered as far user and near user, respectively, in the context of NOMA. Here, we denote the BS as *S*. Due to the existence of PN, the SN meets interference from primary transmitter (PT) who belongs to the PN [27]. Three users in SN also impact on the primary destinations (PD) in the PN, and $h_{SD},h_{D1},h_{D2}$ indicate links, as in Figure 1. The channel coefficients $h_{1},h_{2}$ are used for link BS–first D2D user and BS–second D2D user, respectively. $I_{P}$ is the interference channel from PT affecting on two D2D users. $g_{k,i}$ is the channel coefficient for D2D transmission (i=1,2). It is denoted that channel *u* has exponential distribution with means $\lambda_{u}$, and $I_{P}∼CN\left(0,N_{0}\xi \right)$. Thus, the secondary transmission node *k* is restricted as [27] $P_{k}\leq \min\left({\bar{P}}_{k},\frac{I_{th}}{{\left|h_{Y}\right|}^{2}}\right)$, $k\in \left\{S,U_{1},U_{2}\right\}$ and $Y\in \left(SD,D1,D2\right)$. ${\bar{P}}_{k}$ stands for the maximum average transmission power and $I_{th}$ indicates the interference temperature constraint (ITC) at PD.

We call $x_{1}$ and $x_{2}$ as the messages sent by the BS, which serve both the weak user $U_{1}$ and the strong user $U_{2}$. $a_{1}$ and $a_{2}$ are the power allocation coefficients in NOMA scheme. Following the principle of NOMA, we assume that $a_{1}>a_{2}$ with $a_{1}+a_{2}=1$.

In the first phase, the received signal at the user $U_{i}$, $i\in \left\{1,2\right\}$ is expressed as,

(1) $$y_{n,i}\left(k\right)=h_{i}\left[\sqrt{P_{S}a_{1}}s_{1}\left(k\right)+\sqrt{P_{S}a_{2}}s_{2}\left(k\right)\right]+I_{P}+n_{i}\left(k\right),$$

where $n_{i}$ stands for the additive white Gaussian noise (AWGN) at the receiver with variance $N_{0}$. Regarding detection, the signal-to-interference-plus-noise ratio (SINR) after detecting $s_{1}$ of user $U_{1}$ can be computed as,

(2) $$\gamma_{1,1}=\frac{\rho_{S}a_{1}{\left|h_{1}\right|}^{2}}{\rho_{S}a_{2}{\left|h_{1}\right|}^{2}+\left(\xi +1\right)},$$

where $\rho_{S}=\frac{P_{S}}{N_{0}}$ is the transmission SNR at the BS. Similarly, the SINR to decode signal $s_{1}$ at user $U_{2}$ is given by,

(3) $$\gamma_{2,1}=\frac{\rho_{S}a_{1}{\left|h_{2}\right|}^{2}}{\rho_{S}a_{2}{\left|h_{2}\right|}^{2}+\left(\xi +1\right)}.$$

To implement NOMA, by performing successive interference cancellation (SIC), it can be determined to detect signal $x_{2}$ at $U_{2}$, and we have signal-to-noise (SNR) as,

(4) $$\gamma_{2,2}=\frac{\rho_{S}a_{2}{\left|h_{2}\right|}^{2}}{\left(\xi +1\right)}.$$

## 3. Scheme 1: Performance Analysis of Outage Probability without D2D Link

### 3.1. Outage Probability of $U_{1}$

In this section, the performance analysis of outage probability (OP) without D2D link is investigated. We call this as Scheme 1. The OP is calculated based on the probability density function (PDF) and the cumulative distribution function (CDF) of channel $g_{k,i}$, which can be represented as,

(5) $$f_{{\left|g_{k,i}\right|}^{2}}\left(x\right)=\frac{1}{\lambda_{k,i}}e^{-\frac{x}{\lambda_{k,i}}},$$

and

(6) $$F_{{\left|g_{k,i}\right|}^{2}}\left(x\right)=1-e^{-\frac{x}{\lambda_{k,i}}}.$$

The outage probability (OP) of the message is defined as the probability that the achievable SNR is below a predefined SNR. In this case, we denote $\varepsilon_{1}=2^{2R_{1}}-1$, and $R_{1}$ is the target rate for user $U_{1}$. By denoting Pr(.) as the probability function, the outage behavior of user $U_{1}$ can be shown as,

(7) $$OP_{U_{1}}^{I}=1-\mathrm{Pr}\left\{\gamma_{1,1}>\varepsilon_{1}\right\}.$$

**Proposition** **1.** *The outage probability of $U_{1}$ is given by,*

(8) $$OP_{U_{1}}^{I}=1-\left[e^{-\frac{\theta }{{\bar{\rho }}_{S}\lambda_{1}}}\left(1-e^{-\frac{\rho_{I}}{{\bar{\rho }}_{S}\lambda_{SP}}}\right)+\ell_{1}e^{-\left(\frac{\rho_{I}\lambda_{1}+\lambda_{SP}\theta }{{\bar{\rho }}_{S}\lambda_{1}\lambda_{SP}}\right)}\right],$$

*where $\theta =\frac{\upsilon }{\left(a_{1}-\varepsilon_{1}a_{2}\right)}$, $\upsilon =\varepsilon_{1}\left(\xi +1\right)$ and $\ell_{1}=\frac{\lambda_{1}\rho_{I}}{\lambda_{SP}\theta +\lambda_{1}\rho_{I}}$.*

**Proof.**  See Appendix A. □

### 3.2. Outage Probability of $U_{2}$

It is worth noting that the successful operation of $U_{2}$ happens based on SIC. Therefore, the outage probability of $U_{2}$ is expressed as

(9) $$OP_{U_{2}}^{I}=1-\mathrm{Pr}\left\{\gamma_{2,1}>\varepsilon_{1},\gamma_{2,2}>\varepsilon_{2}\right\}.$$

Similar to solving expression obtained from Equation (7), it can be formulated that

(10) $$\begin{array}{c} OP_{U_{2}}^{I}=1-\left[\mathrm{Pr}\left\{{\left|h_{2}\right|}^{2}>\frac{\psi }{{\bar{\rho }}_{S}},{\left|h_{SP}\right|}^{2}<\frac{\rho_{I}}{{\bar{\rho }}_{S}}\right\}\right.\\ \left.+\mathrm{Pr}\left\{{\left|h_{2}\right|}^{2}>\frac{\psi }{\rho_{I}}{\left|h_{SP}\right|}^{2},{\left|h_{SP}\right|}^{2}>\frac{\rho_{I}}{{\bar{\rho }}_{S}}\right\}\right], \end{array}$$

where $\varepsilon_{2}=2^{2R_{2}}-1$, $\upsilon_{2}=\varepsilon_{2}\left(\xi +1\right)$ and $\psi =\max\left(\frac{\upsilon_{2}}{a_{2}},\theta \right)$. Thus, it can be rewritten in the following form after some manipulations:

(11) $$OP_{U_{2}}^{I}=1-\left[e^{-\frac{\psi }{{\bar{\rho }}_{S}\lambda_{2}}}\left(1-e^{-\frac{\rho_{I}}{{\bar{\rho }}_{S}\lambda_{SP}}}\right)+\frac{\lambda_{2}\rho_{I}e^{-\frac{\rho_{I}\lambda_{2}+\psi \lambda_{SP}}{{\bar{\rho }}_{S}\lambda_{2}\lambda_{SP}}}}{\left(\lambda_{2}\rho_{I}+\psi \lambda_{SP}\right)}\right].$$

**Remark** **1.** *In the context of NOMA architecture, the two paired users’ demand signals are superposed based on difference in their channel conditions and they acquire the same frequency band. In a real deployment, the hardware designer needs to know the quality of such CR-NOMA adapting to signal detection at each receiver. In this regard, outage probabilities in Equations (8) and (11) are crucial terms the need to be studied in terms of system performance. As a result, the outage performance is an important metric to evaluate the success of the transmission with the existence of D2D link at secondary network in such a CR-NOMA. As with most recent works, outage probability is a priority evaluation in these investigations to highlight advantages of emerging techniques [11,12,13,14,15,16,17,18,19,20,21]. Due to the high complexity for the two derived expressions for $U_{1}$ and $U_{2}$, numerical simulations are presented in the next section.*

### 3.3. Asymptotic Analysis

To provide further insight, asymptotic outage performance is presented.

First, we consider the case of ${\bar{\rho }}_{S}\to \infty$, and then approximate outage behaviors for users $U_{1},U_{2}$ are computed.

(12) $$OP_{U_{1}}^{\;{I,\bar{\rho }}_{S}\to \infty }=1-\ell_{1},$$

and

(13) $$OP_{U_{2}}^{\;{I,\bar{\rho }}_{S}\to \infty }\approx 1-\frac{\lambda_{2}}{\left(\lambda_{2}+\bar{\psi }\lambda_{SP}\right)}.$$

Second, in the case of $\rho_{I}\to \infty$, approximate outage behaviors for users $U_{1},U_{2}$ are given as,

(14) $$OP_{U_{1}}^{\;I,\rho_{I}\to \infty }=1-e^{-\frac{\theta }{{\bar{\rho }}_{S}\lambda_{1}}},$$

and

(15) $$OP_{U_{2}}^{\;I,\rho_{I}\to \infty }=1-e^{-\frac{\psi }{{\bar{\rho }}_{S}\lambda_{2}}}.$$

## 4. Scheme 2: Performance Analysis of Outage Probability with D2D Link

Now, we present the outage probability with D2D Link. We call this as Scheme 2. $s_{c}$ is a signal which communicates via such a D2D link. Then, the received signal at user $U_{i}$ can be given as,

(16) $$c_{i}=g_{k,i}P_{U_{i}}s_{c}+I_{P}+n_{c,i},$$

where i≠k. Here, we denote $g_{k,i}$ as a Rayleigh fading channel coefficient in D2D link from user *k* to user *i*, and $s_{c},s_{1}$ are unit signals with $E\left\{{\left|s_{1}\right|}^{2}\right\}=E\left\{{\left|s_{c}\right|}^{2}\right\}=1$. In the next step, SNR can be obtained to decode a signal at each user corresponding to the D2D link,

(17) $$W_{i}=\frac{{\left|g_{k,i}\right|}^{2}P_{U_{i}}}{N_{0}}=\frac{\rho_{U_{i}}{\left|g_{k,i}\right|}^{2}}{\left(\xi +1\right)},$$

where $\rho_{U_{i}}=\frac{P_{U_{i}}}{N_{0}}$. In final step, the SINR for decoding $s_{1}$ under a combination of the D2D link and downlink where NOMA from the BS is given by,

(18) $$Z_{\mathrm{cN},1}^{\mathrm{bi}}=\left\{\begin{array}{cc} \min\left(\max\left\{\gamma_{1,1},W_{1}\right\},\gamma_{2,1}\right) & if\;\;{\left|h_{1}\right|}^{2}<{\left|h_{2}\right|}^{2},\\ \min\left(\max\left\{\gamma_{2,1},W_{2}\right\},\gamma_{1,1}\right) & otherwise \end{array}\right.$$

### 4.1. Outage Probability of $U_{1}$

The outage probability (OP) related to the message at user $U_{i}$ is defined as the probability that the SINR is below a predefined SINR $\varepsilon_{i}$. If the non-SIC user ($U_{1}$) meets the outage behavior, the SIC user ($U_{2}$) does not require a signal from the D2D link. In addition, the outage of the SIC user does not allow the cooperation from the D2D link. Then, we compute the OP for $U_{1}$ as,

(19) $$\begin{array}{cc} OP_{U_{1}}^{II}= & \underset{A_{1}}{\underbrace{\mathrm{Pr}\left(\gamma_{1,1}<\varepsilon_{1},\gamma_{2,1}<\varepsilon_{1}\right)}}\\ & +\underset{A_{2}}{\underbrace{\mathrm{Pr}\left(\max\left(\gamma_{1,1},W_{1}\right)<\varepsilon_{1},\gamma_{2,1}>\varepsilon_{1}\right)}} \end{array}$$

**Lemma** **1.** *The expected $A_{1}$ is given by*

(20) $$\begin{array}{c} A_{1}=\left(1-e^{\frac{\theta }{\lambda_{1}{\bar{\rho }}_{S}}}\right)\left(1-e^{\frac{\theta }{\lambda_{2}{\bar{\rho }}_{S}}}\right)\left(1-e^{-\frac{\rho_{I}}{\lambda_{SP}{\bar{\rho }}_{S}}}\right)\\ +e^{-\frac{\rho_{I}}{{\bar{\rho }}_{S}\lambda_{SP}}}-\ell_{1}e^{-\frac{\lambda_{SP}\theta +\lambda_{1}\rho_{I}}{\lambda_{SP}\lambda_{1}{\bar{\rho }}_{S}}}-\ell_{2}e^{-\frac{\lambda_{SP}\theta +\lambda_{2}\rho_{I}}{\lambda_{SP}\lambda_{2}{\bar{\rho }}_{S}}}\\ +\ell_{3}e^{-\frac{\lambda_{SP}\lambda_{2}\theta +\lambda_{SP}\lambda_{1}\theta +\lambda_{1}\lambda_{2}\rho_{I}}{\lambda_{SP}\lambda_{1}\lambda_{2}{\bar{\rho }}_{S}}} \end{array},$$

*where $\ell_{2}=\frac{\lambda_{2}\rho_{I}}{\lambda_{SP}\theta +\lambda_{2}\rho_{I}}$ and $\ell_{3}=\frac{\lambda_{1}\lambda_{2}\rho_{I}}{\lambda_{SP}\lambda_{2}\theta +\lambda_{SP}\lambda_{1}\theta +\lambda_{1}\lambda_{2}\rho_{I}}$.*

**Proof.**  See Appendix B. □

Then, $A_{2}$ can be expressed as,

(21) $$\begin{array}{cc} A_{2}= & \mathrm{Pr}\left(\max\left(\gamma_{1,1},W_{1}\right)<\varepsilon_{1},\gamma_{2,1}>\varepsilon_{1}\right)\\ = & \underset{A_{2,1}}{\underbrace{\mathrm{Pr}\left(\gamma_{1,1}<\varepsilon_{1},W_{1}<\varepsilon_{1}\right)}}\underset{A_{2,2}}{\underbrace{\mathrm{Pr}\left(\gamma_{2,1}>\varepsilon_{1}\right)}} \end{array}$$

In this study, $R_{1},R_{2}$ denote the target rates corresponding to users $U_{1},U_{2}$.

Next, $A_{2,1}$ can be formulated as,

(22) $$A_{2,1}=\mathrm{Pr}\left(\gamma_{1,1}<\varepsilon_{1}\right)\mathrm{Pr}\left(W_{1}<\varepsilon_{1}\right),$$

Conditioning on $h_{SP}$, $\mathrm{Pr}\left(\gamma_{1,1}<\varepsilon_{1}\right)$ can be calculated as,

(23) $$\begin{array}{c} \mathrm{Pr}\left(\gamma_{1,1}<\varepsilon_{1}\right)=\mathrm{Pr}\left({\left|h_{1}\right|}^{2}<\frac{\theta }{{\bar{\rho }}_{S}},{\left|h_{SP}\right|}^{2}<\frac{\rho_{I}}{{\bar{\rho }}_{S}}\right)+\\ \mathrm{Pr}\left({\left|h_{1}\right|}^{2}<\frac{{\left|h_{SP}\right|}^{2}\theta }{\rho_{I}},{\left|h_{SP}\right|}^{2}>\frac{\rho_{I}}{{\bar{\rho }}_{S}}\right) \end{array}$$

Then, $\mathrm{Pr}\left(\gamma_{1,1}<\varepsilon_{1}\right)$ can be rewritten as,

(24) $$\begin{array}{c} \mathrm{Pr}\left(\gamma_{1,1}<\varepsilon_{1}\right)=\left(1-e^{-\frac{\theta }{\lambda_{1}{\bar{\rho }}_{S}}}\right)\left(1-e^{-\frac{\rho_{I}}{\lambda_{SP}{\bar{\rho }}_{S}}}\right)\\ +e^{-\frac{\rho_{I}}{\lambda_{SP}{\bar{\rho }}_{S}}}-\ell_{1}e^{-\frac{\theta \lambda_{SP}+\lambda_{1}\rho_{I}}{\lambda_{1}{\bar{\rho }}_{S}\lambda_{SP}}} \end{array}$$

The second term of Equation (22) is calculated by,

(25) $$\begin{array}{c} \mathrm{Pr}\left(W_{1}<\varepsilon_{1}\right)=\left({\left|g_{k,1}\right|}^{2}<\frac{\upsilon }{{\bar{\rho }}_{U}},{\left|h_{D1}\right|}^{2}<\frac{\rho_{I}}{{\bar{\rho }}_{U}}\right)\\ +\mathrm{Pr}\left({\left|g_{k,1}\right|}^{2}<\frac{\upsilon {\left|h_{D1}\right|}^{2}}{\rho_{I}},{\left|h_{D1}\right|}^{2}>\frac{\rho_{I}}{{\bar{\rho }}_{U}}\right)\\ =\left(1-e^{-\frac{\upsilon }{\lambda_{k,1}{\bar{\rho }}_{U}}}\right)\left(1-e^{-\frac{\rho_{I}}{\lambda_{D1}{\bar{\rho }}_{U}}}\right)\\ +e^{-\frac{\rho_{I}}{\lambda_{SP}{\bar{\rho }}_{U}}}-\ell_{4}e^{-\frac{\upsilon \lambda_{SP}+\lambda_{k,1}\rho_{I}}{\lambda_{k,1}{\bar{\rho }}_{S}\lambda_{SP}}} \end{array}$$

where $\ell_{4}=\frac{\lambda_{k,1}\rho_{I}}{\upsilon \lambda_{SP}+\lambda_{k,1}\rho_{I}}$.

Similarly, $A_{2,2}$ can be obtained as,

(26) $$\begin{array}{cc} A_{2,2}= & \mathrm{Pr}\left({\left|h_{2}\right|}^{2}>\frac{\theta }{{\bar{\rho }}_{S}},{\left|h_{SP}\right|}^{2}<\frac{\rho_{I}}{{\bar{\rho }}_{S}}\right)\\ & +\mathrm{Pr}\left({\left|h_{2}\right|}^{2}>\frac{\theta {\left|h_{S_{n}P}\right|}^{2}}{\rho_{I}},{\left|h_{SP}\right|}^{2}>\frac{\rho_{I}}{{\bar{\rho }}_{S}}\right). \end{array}$$

This can be rewritten as,

(27) $$\begin{array}{cc} A_{2,2}= & e^{-\frac{\theta }{\lambda_{2}{\bar{\rho }}_{S}}}\left(1-e^{-\frac{\rho_{I}}{\lambda_{SP}{\bar{\rho }}_{S}}}\right)+\ell_{2}e^{-\frac{\lambda_{SP}\theta +\lambda_{2}\rho_{I}}{\lambda_{SP}\lambda_{2}{\bar{\rho }}_{S}}}. \end{array}$$

Putting Equations (24), (25) and (27) into Equation (19) and using result in Lemma 1, the outage probability of $U_{2}$ can be expressed as,

(28) $$\begin{array}{c} \begin{array}{c} OP_{U_{1}}^{II}=\left[\left(1-e^{\frac{\theta }{\lambda_{1}{\bar{\rho }}_{S}}}\right)\left(1-e^{\frac{\theta }{\lambda_{2}{\bar{\rho }}_{S}}}\right)\left(1-e^{-\frac{\rho_{I}}{\lambda_{SP}{\bar{\rho }}_{S}}}\right)+e^{-\frac{\rho_{I}}{{\bar{\rho }}_{S}\lambda_{SP}}}\right.\\ \left.-\ell_{1}e^{-\frac{\lambda_{SP}\theta +\lambda_{1}\rho_{I}}{\lambda_{SP}\lambda_{1}{\bar{\rho }}_{S}}}-\ell_{2}e^{-\frac{\lambda_{SP}\theta +\lambda_{2}\rho_{I}}{\lambda_{SP}\lambda_{2}{\bar{\rho }}_{S}}}+\ell_{3}e^{-\frac{\lambda_{SP}\lambda_{2}\theta +\lambda_{SP}\lambda_{1}\theta +\lambda_{1}\lambda_{2}\rho_{I}}{\lambda_{SP}\lambda_{1}\lambda_{2}{\bar{\rho }}_{S}}}\right]+\\ \left(\left(1-e^{-\frac{\theta }{\lambda_{1}{\bar{\rho }}_{S}}}\right)\left(1-e^{-\frac{\rho_{I}}{\lambda_{SP}{\bar{\rho }}_{S}}}\right)+e^{-\frac{\rho_{I}}{\lambda_{SP}{\bar{\rho }}_{S}}}-\ell_{1}e^{-\frac{\theta \lambda_{SP}+\lambda_{1}\rho_{I}}{\lambda_{1}{\bar{\rho }}_{S}\lambda_{SP}}}\right)\\ \times \left(\left(1-e^{-\frac{\upsilon }{\lambda_{k,1}{\bar{\rho }}_{U}}}\right)\left(1-e^{-\frac{\rho_{I}}{\lambda_{D1}{\bar{\rho }}_{U}}}\right)+e^{-\frac{\rho_{I}}{\lambda_{SP}{\bar{\rho }}_{U}}}-\ell_{4}e^{-\frac{\upsilon \lambda_{SP}+\lambda_{k,1}\rho_{I}}{\lambda_{k,1}{\bar{\rho }}_{S}\lambda_{SP}}}\right)\\ \times \left(e^{-\frac{\theta }{\lambda_{2}{\bar{\rho }}_{S}}}\left(1-e^{-\frac{\rho_{I}}{\lambda_{SP}{\bar{\rho }}_{S}}}\right)+\ell_{2}e^{-\frac{\lambda_{SP}\theta +\lambda_{2}\rho_{I}}{\lambda_{SP}\lambda_{2}{\bar{\rho }}_{S}}}\right). \end{array} \end{array}$$

### 4.2. Outage Probability of $U_{2}$

Similarly, the outage probability of $U_{2}$ can be formulated as,

(29) $$\begin{array}{c} \begin{array}{c} OP_{U_{2}}^{II}=\underset{B_{1}}{\underbrace{\mathrm{Pr}\left\{\left(\gamma_{2,2}<\varepsilon_{2}\cup \gamma_{2,1}<\varepsilon_{1}\right),\gamma_{1,1}<\varepsilon_{1}\right\}}}\\ +\underset{B_{2}}{\underbrace{\mathrm{Pr}\left\{\left(\gamma_{2,2}<\varepsilon_{2}\cup \max\left(\gamma_{2,1},W_{2}\right)<\varepsilon_{1}\right),\gamma_{1,1}>\varepsilon_{1}\right\}}} \end{array} \end{array}$$

Then, $B_{1}$ is computed by,

(30) $$\begin{array}{c} \begin{array}{cc} B_{1}= & \mathrm{Pr}\left\{\left(\gamma_{2,2}<\varepsilon_{2}\cup \gamma_{2,1}<\varepsilon_{1}\right),\gamma_{1,1}<\varepsilon_{1}\right\}\\ = & \underset{B_{1,1}}{\underbrace{\left[1-\mathrm{Pr}\left\{\left(\gamma_{2,2}>\varepsilon_{2},\gamma_{2,1}>\varepsilon_{1}\right)\right\}\right]}}\underset{B_{1,2}}{\underbrace{\mathrm{Pr}\left\{\gamma_{1,1}<\varepsilon_{1}\right\}}} \end{array} \end{array}$$

Similarly, $B_{1,1}$ can be achieved as

(31) $$\begin{array}{c} \begin{array}{c} B_{1,1}=1-\left[\mathrm{Pr}\left({\left|h_{2}\right|}^{2}>\frac{\psi }{{\bar{\rho }}_{S}},{\left|h_{SP}\right|}^{2}<\frac{\rho_{I}}{{\bar{\rho }}_{S}}\right)\right.\\ +\left.\mathrm{Pr}\left({\left|h_{2}\right|}^{2}>\frac{\psi {\left|h_{SP}\right|}^{2}}{\rho_{I}},{\left|h_{SP}\right|}^{2}>\frac{\rho_{I}}{{\bar{\rho }}_{S}}\right)\right]\\ =1-\left\{e^{-\frac{\psi }{\lambda_{2}{\bar{\rho }}_{S}}}\left(1-e^{-\frac{\rho_{I}}{\lambda_{SP}{\bar{\rho }}_{S}}}\right)+\frac{\lambda_{2}\rho_{I}e^{-\frac{\psi \lambda_{SP}+\lambda_{2}\rho_{I}}{\lambda_{SP}\lambda_{2}{\bar{\rho }}_{S}}}}{\psi \lambda_{SP}+\lambda_{2}\rho_{I}}\right\}. \end{array} \end{array}$$

In a similar way, $B_{1,2}$ can be written as

(32) $$\begin{array}{c} \begin{array}{c} B_{1,2}=\left(1-e^{-\frac{\theta }{\lambda_{1}{\bar{\rho }}_{S}}}\right)\left(1-e^{-\frac{\rho_{I}}{\lambda_{SP}{\bar{\rho }}_{S}}}\right)\\ +\left(e^{-\frac{\rho_{I}}{\lambda_{SP}{\bar{\rho }}_{S}}}-\ell_{1}e^{-\frac{\theta \lambda_{SP}+\lambda_{1}\rho_{I}}{\lambda_{SP}\lambda_{1}{\bar{\rho }}_{S}}}\right) \end{array} \end{array}$$

As a result, $B_{1}$ can be computed as follows.

(33) $$\begin{array}{c} \begin{array}{cc} B_{1}= & \left\{1-\left[e^{-\frac{\psi }{\lambda_{2}{\bar{\rho }}_{S}}}\left(1-e^{-\frac{\rho_{I}}{\lambda_{SP}{\bar{\rho }}_{S}}}\right)+\frac{\lambda_{2}\rho_{I}e^{-\frac{\psi \lambda_{SP}+\lambda_{2}\rho_{I}}{\lambda_{SP}\lambda_{2}{\bar{\rho }}_{S}}}}{\psi \lambda_{SP}+\lambda_{2}\rho_{I}}\right]\right\}\\ \times & \left(\left(1-e^{-\frac{\theta }{\lambda_{1}{\bar{\rho }}_{S}}}\right)\left(1-e^{-\frac{\rho_{I}}{\lambda_{SP}{\bar{\rho }}_{S}}}\right)+\left(e^{-\frac{\rho_{I}}{\lambda_{SP}{\bar{\rho }}_{S}}}-\ell_{1}e^{-\frac{\theta \lambda_{SP}+\lambda_{1}\rho_{I}}{\lambda_{SP}\lambda_{1}{\bar{\rho }}_{S}}}\right)\right) \end{array}. \end{array}$$

Likewise, following results can be achieved for $B_{2}$.

(34) $$\begin{array}{c} \begin{array}{cc} B_{2}= & \mathrm{Pr}\left\{\left(\gamma_{2,2}<\varepsilon_{2}\cup \max\left(\gamma_{2,1},W_{2}\right)<\varepsilon_{1}\right),\gamma_{1,1}>\varepsilon_{1}\right\}\\ = & \left[B_{2,1}+B_{2,2}-B_{2,3}\right]B_{2,4} \end{array}. \end{array}$$

where $B_{2,3}=\mathrm{Pr}\left\{\gamma_{2,2}<\varepsilon_{2},\gamma_{2,1}<\varepsilon_{1},W_{2}<\varepsilon_{1}\right\}$, $B_{2,1}=\mathrm{Pr}\left\{\gamma_{2,2}<\varepsilon_{2}\right\}$, $B_{2,2}=\mathrm{Pr}\left\{\max\left(\gamma_{2,1},W_{2}\right)<\varepsilon_{1}\right\}$ and $B_{2,4}=\mathrm{Pr}\left\{\gamma_{1,1}>\varepsilon_{1}\right\}$. More specifically, $B_{2,1}$ is given as

(35) $$\begin{array}{c} \begin{array}{cc} B_{2,1}= & \left(1-e^{-\frac{\upsilon_{2}}{\lambda_{2}{\bar{\rho }}_{S}\Theta_{2}}}\right)\left(1-e^{-\frac{\rho_{I}}{\lambda_{SP}{\bar{\rho }}_{S}}}\right)+e^{-\frac{\rho_{I}}{\lambda_{SP}{\bar{\rho }}_{S}}}\\ & -\frac{\lambda_{2}\rho_{I}\Theta_{2}}{\lambda_{SP}\upsilon_{2}+\lambda_{2}\rho_{I}\Theta_{2}}e^{-\frac{\lambda_{SP}\upsilon_{2}+\lambda_{2}\rho_{I}\Theta_{2}}{\lambda_{SP}\lambda_{2}{\bar{\rho }}_{S}\Theta_{2}}}. \end{array} \end{array}$$

Next, $B_{2,2}$ can be calculated as

(36) $$\begin{array}{c} \begin{array}{c} B_{2,2}=\left(\left(1-e^{-\frac{\theta }{\lambda_{2}{\bar{\rho }}_{S}}}\right)\left(1-e^{-\frac{\rho_{I}}{\lambda_{SP}{\bar{\rho }}_{S}}}\right)\right.+e^{-\frac{\rho_{I}}{\lambda_{SP}{\bar{\rho }}_{S}}}\\ -\ell_{2}e^{-\frac{\theta \lambda_{SP}+\lambda_{2}\rho_{I}}{\lambda_{2}{\bar{\rho }}_{S}\lambda_{SP}}}\times \left(\left(1-e^{-\frac{\upsilon }{\lambda_{k,2}{\bar{\rho }}_{U}}}\right)\left(1-e^{-\frac{\rho_{I}}{\lambda_{D2}{\bar{\rho }}_{U}}}\right)\right.\\ \left.+e^{-\frac{\rho_{I}}{\lambda_{SP}{\bar{\rho }}_{U}}}-\ell_{4}e^{-\frac{\upsilon \lambda_{SP}+\lambda_{k,2}\rho_{I}}{\lambda_{k,2}{\bar{\rho }}_{S}\lambda_{SP}}}\right). \end{array} \end{array}$$

$B_{2,3}$ and $B_{2,4}$ need to be computed, and they can be given as

(37) $$\begin{array}{c} \begin{array}{c} B_{2,3}=\left(\left(1-e^{-\frac{\varpi }{\lambda_{2}{\bar{\rho }}_{S}}}\right)\left(1-e^{-\frac{\rho_{I}}{\lambda_{SP}{\bar{\rho }}_{S}}}\right)+e^{-\frac{\rho_{I}}{\lambda_{SP}{\bar{\rho }}_{S}}}\right.\\ -\frac{\lambda_{2}\rho_{I}e^{-\frac{\varpi \lambda_{SP}+\lambda_{2}\rho_{I}}{\lambda_{2}{\bar{\rho }}_{S}\lambda_{SP}}}}{\varpi \lambda_{SP}+\lambda_{2}\rho_{I}}\times \left(\left(1-e^{-\frac{\upsilon }{\lambda_{k,2}{\bar{\rho }}_{U}}}\right)\left(1-e^{-\frac{\rho_{I}}{\lambda_{D2}{\bar{\rho }}_{U}}}\right)\right.\\ \left.+e^{-\frac{\rho_{I}}{\lambda_{SP}{\bar{\rho }}_{U}}}-\ell_{4}e^{-\frac{\upsilon \lambda_{SP}+\lambda_{k,2}\rho_{I}}{\lambda_{k,2}{\bar{\rho }}_{S}\lambda_{SP}}}\right), \end{array} \end{array}$$

(38) $$\begin{array}{c} B_{2,4}=e^{-\frac{\theta }{\lambda_{1}{\bar{\rho }}_{S}}}\left(1-e^{-\frac{\rho_{I}}{\lambda_{SP}{\bar{\rho }}_{S}}}\right)+\ell_{1}e^{-\frac{\lambda_{SP}\theta +\lambda_{1}\rho_{I}}{\lambda_{SP}\lambda_{1}{\bar{\rho }}_{S}}} \end{array}$$

where $\varpi =\min\left(\frac{\upsilon_{2}}{a_{2}},\theta \right)$.

Plugging Equations (34) and (35), into Equation (30), the outage probability of $U_{2}$ can be expressed as

(39) $$\begin{array}{c} OP_{U_{2}}^{II}=\left\{1-\left\{e^{-\frac{\psi }{\lambda_{2}{\bar{\rho }}_{S}}}\left(1-e^{-\frac{\rho_{I}}{\lambda_{SP}{\bar{\rho }}_{S}}}\right)+\frac{\lambda_{2}\rho_{I}e^{-\frac{\psi \lambda_{SP}+\lambda_{2}\rho_{I}}{\lambda_{SP}\lambda_{2}{\bar{\rho }}_{S}}}}{\psi \lambda_{SP}+\lambda_{2}\rho_{I}}\right\}\right\}\times \left(\left(1-e^{-\frac{\theta }{\lambda_{1}{\bar{\rho }}_{S}}}\right)\left(1-e^{-\frac{\rho_{I}}{\lambda_{SP}{\bar{\rho }}_{S}}}\right)\right.\\ \left.+\left(e^{-\frac{\rho_{I}}{\lambda_{SP}{\bar{\rho }}_{S}}}-\ell_{1}e^{-\frac{\theta \lambda_{SP}+\lambda_{1}\rho_{I}}{\lambda_{SP}\lambda_{1}{\bar{\rho }}_{S}}}\right)\right)+\left\{\left(1-e^{-\frac{\upsilon_{2}}{\lambda_{2}{\bar{\rho }}_{S}a_{2}}}\right)\left(1-e^{-\frac{\rho_{I}}{\lambda_{SP}{\bar{\rho }}_{S}}}\right)+e^{-\frac{\rho_{I}}{\lambda_{SP}{\bar{\rho }}_{S}}}-\frac{\lambda_{2}\rho_{I}a_{2}e^{-\frac{\lambda_{SP}\upsilon_{2}+\lambda_{2}\rho_{I}a_{2}}{\lambda_{SP}\lambda_{2}{\bar{\rho }}_{S}a_{2}}}}{\lambda_{SP}\upsilon_{2}+\lambda_{2}\rho_{I}a_{2}}\right.\\ +\left(\left(1-e^{-\frac{\theta }{\lambda_{2}{\bar{\rho }}_{S}}}\right)\left(1-e^{-\frac{\upsilon }{\lambda_{k,2}{\bar{\rho }}_{U}}}\right)\left(1-e^{-\frac{\rho_{I}}{\lambda_{SP}{\bar{\rho }}_{S}}}\right)\left(1-e^{-\frac{\rho_{I}}{\lambda_{D2}{\bar{\rho }}_{U}}}\right)\right.+\left(e^{-\frac{\rho_{I}}{\lambda_{D2}{\bar{\rho }}_{U}}}-\frac{\lambda_{k,2}\rho_{I}e^{-\frac{\lambda_{D2}\upsilon +\lambda_{k,2}\rho_{I}}{\lambda_{D2}\lambda_{k,2}{\bar{\rho }}_{U}}}}{\lambda_{D2}\upsilon +\lambda_{k,2}\rho_{I}}\right)\\ \times \left.\left(e^{-\frac{\rho_{I}}{{\bar{\rho }}_{S}\lambda_{SP}}}-\frac{\lambda_{2}\rho_{I}e^{-\frac{\lambda_{SP}\theta +\lambda_{2}\rho_{I}}{\lambda_{SP}\lambda_{2}{\bar{\rho }}_{S}}}}{\lambda_{SP}\theta +l\lambda_{2}\rho_{I}}\right)\right)-\left(\left(1-e^{-\frac{\varpi }{\lambda_{2}{\bar{\rho }}_{S}}}\right)\left(1-e^{-\frac{\upsilon }{\lambda_{k,2}{\bar{\rho }}_{U}}}\right)\left(1-e^{-\frac{\rho_{I}}{\lambda_{SP}{\bar{\rho }}_{S}}}\right)\left(1-e^{-\frac{\rho_{I}}{\lambda_{D2}{\bar{\rho }}_{U}}}\right)\right.\\ \left.\left.+\left(e^{-\frac{\rho_{I}}{\lambda_{D2}{\bar{\rho }}_{U}}}-\frac{\lambda_{k,2}\rho_{I}}{\lambda_{D2}\upsilon +\lambda_{k,2}\rho_{I}}e^{-\frac{\lambda_{D2}\upsilon +\lambda_{k,2}\rho_{I}}{\lambda_{D2}\lambda_{k,2}{\bar{\rho }}_{U}}}\right)\left(e^{-\frac{\rho_{I}}{{\bar{\rho }}_{S}\lambda_{SP}}}-\frac{\lambda_{2}\rho_{I}}{\lambda_{SP}\varpi +\lambda_{2}\rho_{I}}e^{-\frac{\lambda_{SP}\varpi +\lambda_{2}\rho_{I}}{\lambda_{SP}\lambda_{2}{\bar{\rho }}_{S}}}\right)\right)\right\}\\ \times \left(e^{-\frac{\theta }{\lambda_{1}{\bar{\rho }}_{S}}}\left(1-e^{-\frac{\rho_{I}}{\lambda_{SP}{\bar{\rho }}_{S}}}\right)+\frac{\lambda_{1}\rho_{I}}{\lambda_{SP}\theta +\lambda_{1}\rho_{I}}e^{-\frac{\lambda_{SP}\theta +\lambda_{1}\rho_{I}}{\lambda_{SP}\lambda_{1}{\bar{\rho }}_{S}}}\right). \end{array}$$

**Remark** **2.** *It is difficult to find the optimal outage performance of each user in SN of CR-NOMA. It is expected that the numerical method would provide the optimal value of power allocation factor to indicate the lowest outage performance. The numerical result section introduces optimal performance. Such analysis and related guidelines are useful evaluations before implementing the CR-NOMA in a practical way.*

### 4.3. Asymptotic Analysis

Since the derived expressions are rather complicated, the asymptotic expressions for the outage probability to provide additional insight needs to be investigated.

In the first case for $\rho_{I}\to \infty$, the asymptotic expression for $OP_{U_{1}}$ is given as

(40) $$\begin{array}{c} \begin{array}{c} OP_{U_{1}}^{II,\rho_{I}\to \infty }=\left(1-e^{-\frac{\theta }{\lambda_{1}{\bar{\rho }}_{S}}}\right)\left(1-e^{-\frac{\theta }{\lambda_{2}{\bar{\rho }}_{S}}}\right)\\ +\left(1-e^{-\frac{\theta }{\lambda_{1}{\bar{\rho }}_{S}}}\right)\left(1-e^{-\frac{\upsilon }{\lambda_{k,1}{\bar{\rho }}_{U}}}\right)e^{-\frac{\theta }{\lambda_{2}{\bar{\rho }}_{S}}} \end{array} \end{array}$$

and

(41) $$\begin{array}{c} \begin{array}{c} OP_{U_{2}}^{II,\rho_{I}\to \infty }=\left\{1-e^{-\frac{\psi }{\lambda_{2}{\bar{\rho }}_{S}}}\right\}\left(1-e^{-\frac{\theta }{\lambda_{1}{\bar{\rho }}_{S}}}\right)\\ +\left(\left(1-e^{-\frac{\upsilon_{2}}{\lambda_{2}{\bar{\rho }}_{S}a_{2}}}\right)\right.+\left(1-e^{-\frac{\theta }{\lambda_{2}{\bar{\rho }}_{S}}}\right)\left(1-e^{-\frac{\upsilon }{\lambda_{k,2}{\bar{\rho }}_{U}}}\right)\\ \left.-\left(1-e^{-\frac{\varpi }{\lambda_{2}{\bar{\rho }}_{S}}}\right)\left(1-e^{-\frac{\upsilon }{\lambda_{k,2}{\bar{\rho }}_{U}}}\right)\right)e^{-\frac{\theta }{\lambda_{1}{\bar{\rho }}_{S}}}. \end{array} \end{array}$$

In the second scenario, i.e., ${\bar{\rho }}_{S}\to \infty$, $OP_{U_{1}}$ is computed as

(42) $$\begin{array}{c} OP_{U_{1}}^{{II,\bar{\rho }}_{S}\to \infty }=\left(1-\ell_{1}-\ell_{2}+\ell_{3}\right)+\left(1-\ell_{1}\right)\left(1-\ell_{4}\right)\left(\ell_{2}\right), \end{array}$$

and

(43) $$\begin{array}{c} \begin{array}{c} OP_{U_{2}}^{{II,\bar{\rho }}_{S}\to \infty }=\left\{1-\frac{\lambda_{2}\rho_{I}}{\psi \lambda_{SP}+\lambda_{2}\rho_{I}}\right\}\left(1-\ell_{1}\right)+\\ \left\{1-\frac{\lambda_{2}\rho_{I}a_{2}}{\lambda_{SP}\upsilon_{2}+\lambda_{2}\rho_{I}a_{2}}\right.+\left(1-\frac{\lambda_{k,2}\rho_{I}}{\lambda_{D2}\upsilon +\lambda_{k,2}\rho_{I}}\right)\left(1-\ell_{2}\right)\\ -\left.\left(1-\frac{\lambda_{k,2}\rho_{I}}{\lambda_{D2}\upsilon +\lambda_{k,2}\rho_{I}}\right)\left(1-\frac{\lambda_{2}\rho_{I}}{\lambda_{SP}\varpi +\lambda_{2}\rho_{I}}\right)\right\}\ell_{1}. \end{array} \end{array}$$

### 4.4. Consideration on Throughput

Regarding other metric n=(I,II), the overall throughput needs to be examined.

(44) $$\begin{array}{c} T_{total}^{n}=R_{1}\left(1-OP_{U_{1}}^{n}\right)+R_{2}\left(1-OP_{U_{2}}^{n}\right) \end{array}$$

## 5. Numerical Results

**Figure 2:**
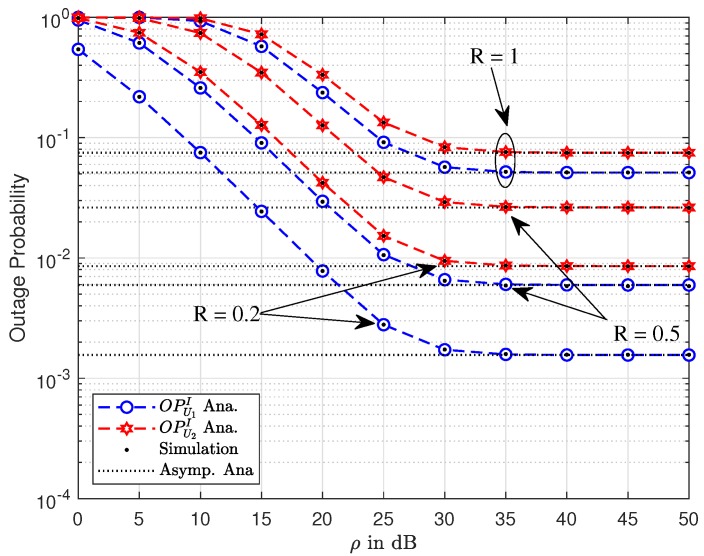
Outage performance of $U_{1}$ and $U_{2}$ versus ρ with varying *R*.

The simulation model is based on Figure 1, and we assume fixed power allocation factors are assigned for two NOMA users: $a_{1}=0.8$ and $a_{2}=0.2$. In the simulations, we set $d_{1}=1.8$, $d_{2}=1.2$, $d_{k,1}=d_{k,2}=3$, $d_{SP}=d_{D1}=d_{D2}=5$, $\rho_{I}=20dB$, $\rho ={\bar{\rho }}_{S}={\bar{\rho }}_{U_{1}}={\bar{\rho }}_{U_{2}}$ and $R=R_{1}=R_{2}=1$ bit per channel use (BPCU). The path loss exponent is χ. Here, we denote “ana.” and “sim.” as analytical results and simulated curves, respectively. MATLAB was employed to verify these derived expressions to further provide system performance evaluation intuitively. Figure 2 shows the OP results obtained for several scenarios of target rates R=0.1,0.5,1. It is easily observed that a higher target rate results in worse outage performance. In addition, the higher ρ converges to the constant value of such OP. The performance gap among two users exists due to the different power allocation factors for each user. We also plot asymptotic cases and lower-bounded approximation, which provides a performance limit of such OP in the proposed system. Furthermore, the analytical results of OP was verified by the simulations. As observed, both match very well.

**Figure 3:**
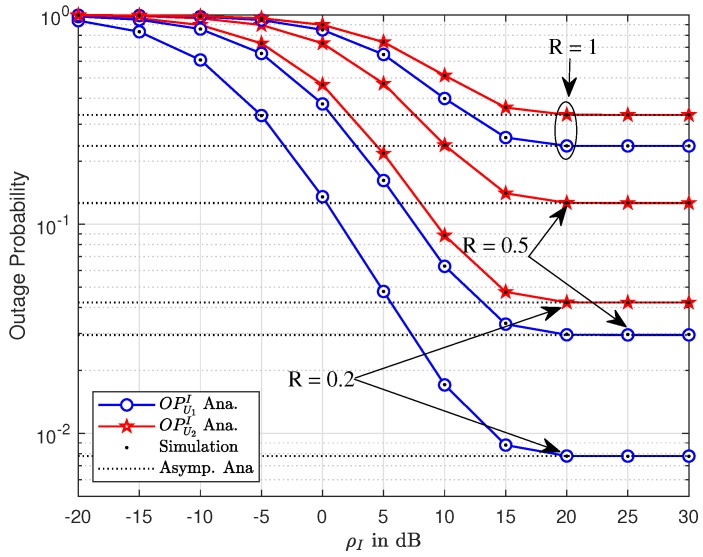
Outage performance of $U_{1}$ and $U_{2}$ versus ${\bar{\rho }}_{S}$ as varying *R*.

In the proposed system model, the impact of interference is indicated in Figure 3. A similar trend of OP can be seen in this illustration compared to Figure 2. Higher target rate and high $\rho_{I}$ exhibit the best outage behavior. Hence, $\rho_{I}=20\;\left(\mathrm{dB}\right),R=0.2\;\left(\mathrm{bps}/\mathrm{Hz}\right)$ is the best outage performance that can be raised in these comparisons.

**Figure 4:**
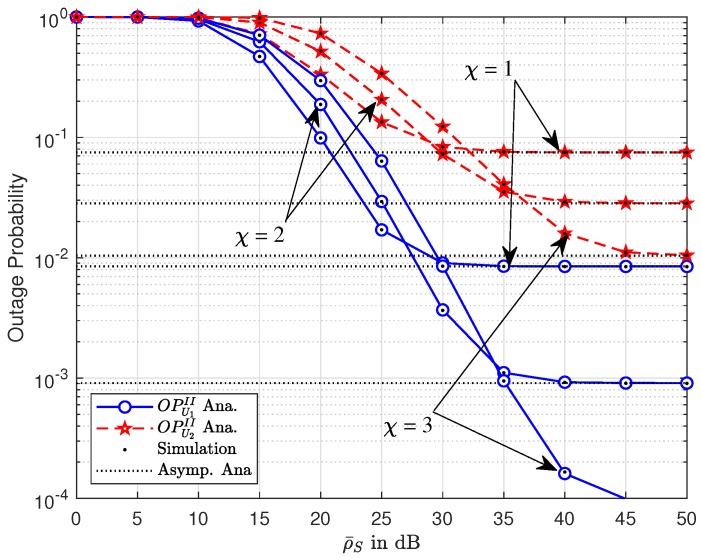
Outage performance of $U_{1}$ and $U_{2}$ versus ${\bar{\rho }}_{S}$ with varying χ.

**Figure 5:**
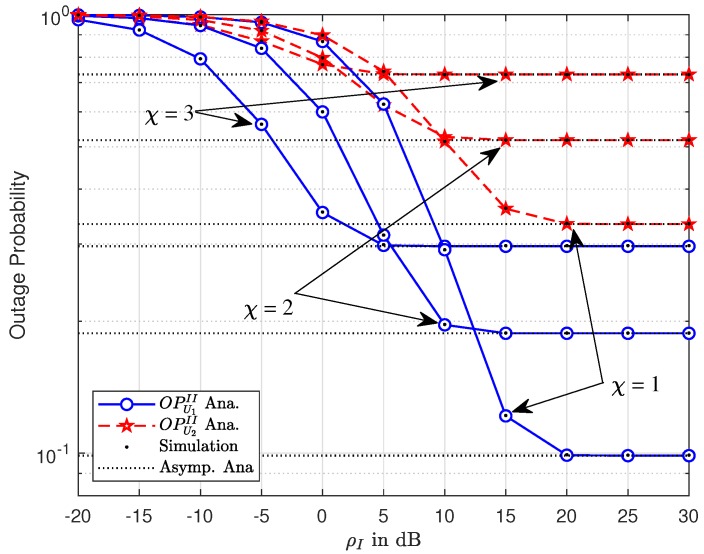
Outage performance of $U_{1}$ and $U_{2}$ versus $\rho_{I}$ with varying χ.

Figure 4 and Figure 5 show the outage performance versus transmission SNR at the BS, ρ and $\rho_{I}$, respectively, to raise the performance gap among two NOMA users in the CR-NOMA scenario with varying path loss exponent factors. It can be seen that the asymptotic curves match the exact curves at high ρ and $\rho_{I}$ for Figure 4 and Figure 5, respectively. Different power allocation factors are recognized as the main reason for such a performance gap. Each figure indicates that outage performance will meet the saturation situation at high ρ and $\rho_{I}$ for Figure 2 and Figure 3, respectively. It can be explained that such result is straightforward from the definition of outage probability. Similar performance with respect to ρ and $\rho_{I}$ can be observed. These figures indicate that there is a good match between the analytical result and the Monte-Carlo simulation result. Such matching observations are also illustrated in the following experiments.

**Figure 6:**
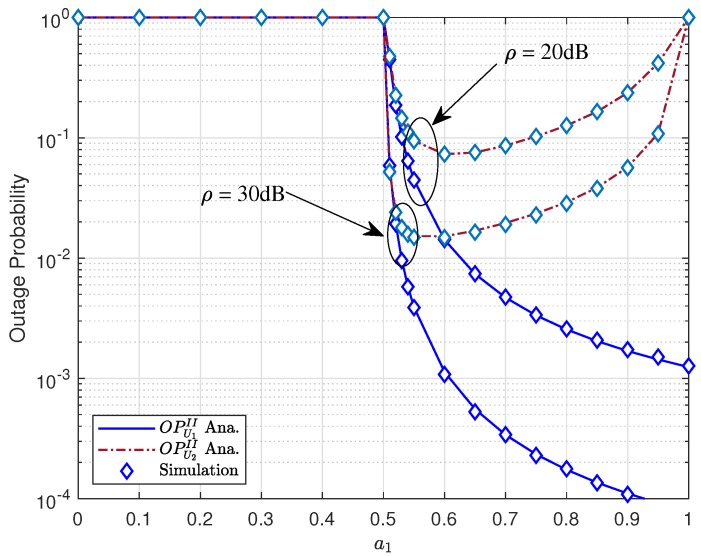
Outage performance of $U_{1}$ and $U_{2}$ versus $a_{1}$.

Figure 6 shows different trends of outage probability of $U_{1},U_{2}$ as increasing power allocation factor $a_{1}$. It is noted that, due to weak users that require higher power allocation factor, it is required that $a_{1}>0.5$. As a result, outage behavior happens as $a_{1}<0.5$ for both users $U_{1}$ and $U_{2}$ in both cases of transmission SNR ρ. In Figure 6, it is noticeable that $a_{1}=1$ leads to outage behavior for $U_{2}$ too. This figure confirms the optimal outage for user $U_{2}$, i.e., optimal power coefficient $a_{1}=0.54$ for ρ=30(dB) and $a_{1}=0.6$ for ρ=20(dB). The reason is that $a_{1}$ contributes to varying SINR, and it makes crucial impact on outage performance.

**Figure 7:**
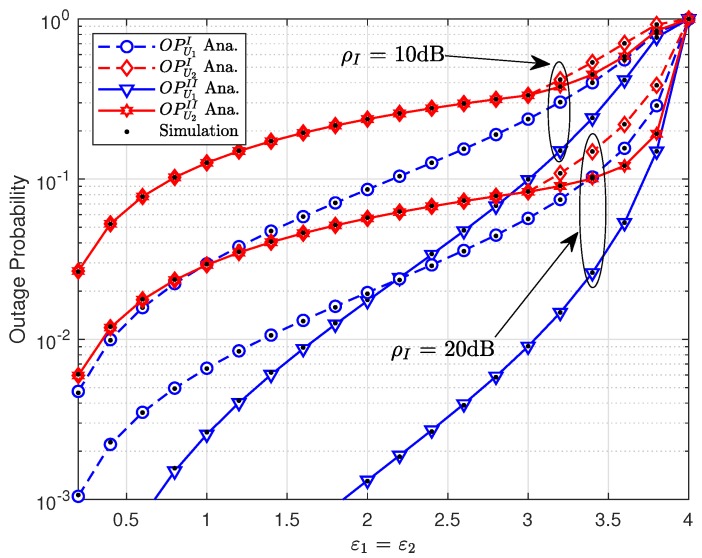
Comparison on two schemes for $U_{1},U_{2}$ with varying the target rate $\epsilon_{1}=\epsilon_{2}$.

To observe the impact of the target rates $R_{1},R_{2}$, Figure 7 indicates the improved performance for both $U_{1},U_{2}$ in Scheme 2 at higher target rates $R_{1},R_{2}$. While the throughput increases too high at highest $R_{1}=R_{2}=1$ among three comparison cases, as in Figure 7, it confirms that the expression of throughput contains both target rates and transmission SNR, hence such throughput performance would change due to the variations of $\rho ,R_{1},R_{2}$. However, when ρ is greater than 25 (dB), it leads to a constant throughput. It can be easily seen that ρ=30(dB) shows the lowest outage performance.

**Figure 8:**
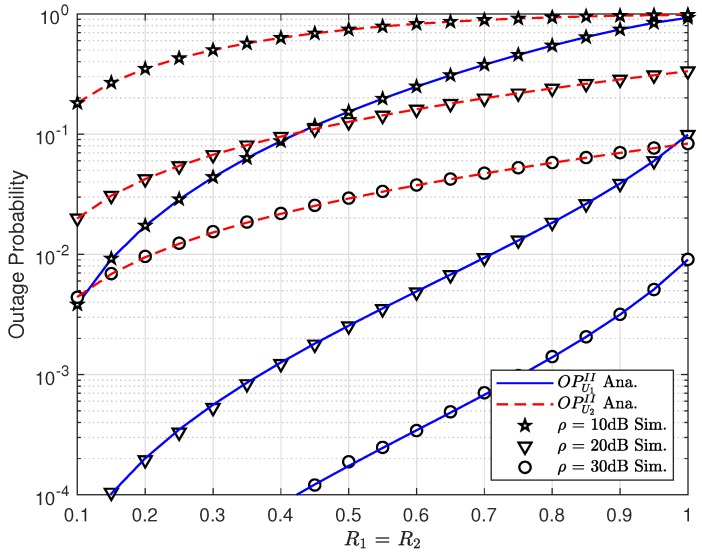
Outage performance of $U_{1}$ and $U_{2}$ versus the target rate $R_{1}=R_{2}$.

We compare outage performance between Scheme 1 and Scheme 2 in Figure 8. In this experiment, we compared two cases of $\rho_{I}$, $\rho_{I}=20\;\left(\mathrm{dB}\right)$ and $\rho_{I}=30\;\left(\mathrm{dB}\right)$. As the previous simulation, the better case is $\rho_{I}=30\;\left(\mathrm{dB}\right)$. It can be confirmed that Scheme 2 is better than Scheme 1 in terms of outage performance at a higher value of $\epsilon_{1}=\epsilon_{2}$, i.e., $\epsilon_{1}=\epsilon_{2}>3$, which shows the performance gap of user $U_{2}$ among the two schemes. However, at a lower value of $\epsilon_{1}=\epsilon_{2}$, performance for $U_{2}$ does not exist. In contrast, we can always find a performance gap among two schemes for $U_{1}$.

**Figure 9:**
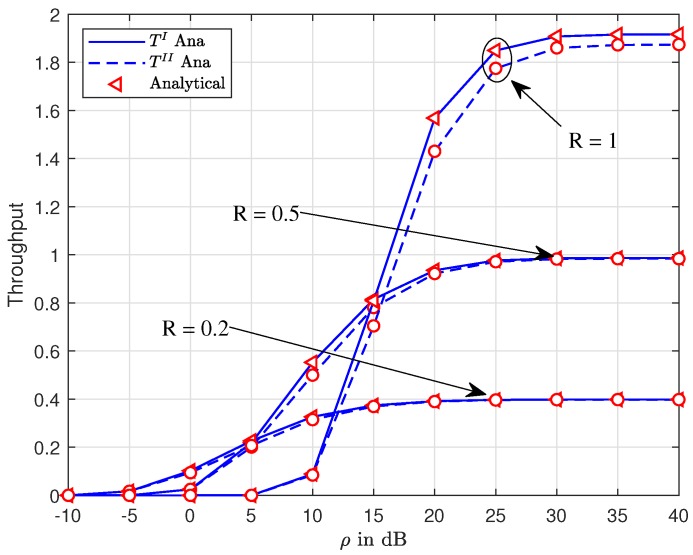
Throughput of system versus ${\bar{\rho }}_{S}$ with varying the target rate $R_{1}=R_{2}$.

Interestingly, the existence of the D2D in the proposed system does not harm the overall throughput performance of both schemes. This can be confirmed in Figure 9. Of course, R=1 shows the highest throughput in the three cases. However, as ρ is greater than 25 (dB), it leads to the saturation value of these throughput curves.

## 6. Conclusions

This paper proposes D2D transmission schemes for CR-NOMA, in which the weak NOMA user cooperates with relay (the strong NOMA user) by exploiting two links. The detailed performance analysis is presented in terms of throughput and outage probability. The closed-form expressions related to these metrics atr derived, and it is shown that a performance gap exists among the two NOMA users. The target rates and power allocation factors give the main impacts on these metrics. In addition, the optimal power allocation factor can be found to obtain optimal outage performance for at least one user in such a NOMA scenario. Simulation results verified the performance analysis, confirming that CR-NOMA works well with the ability of D2D transmission.

## Appendix A

**Proof** **of** **Proposition** **1.** Putting Equation (2) into Equation (7), $OP_{U_{1}}^{I}$ can be expressed as

(A1) $$\begin{array}{c} OP_{U_{1}}^{I}=1-\left[\mathrm{Pr}\left\{{\left|h_{1}\right|}^{2}>\frac{\theta }{{\bar{\rho }}_{S}},{\left|h_{SP}\right|}^{2}<\frac{\rho_{I}}{{\bar{\rho }}_{S}}\right\}\right.\\ \left.+\mathrm{Pr}\left\{{\left|h_{1}\right|}^{2}>\frac{\theta {\left|h_{SP}\right|}^{2}}{\rho_{I}},{\left|h_{SP}\right|}^{2}>\frac{\rho_{I}}{{\bar{\rho }}_{S}}\right\}\right] \end{array}$$

First, $OP_{U_{1}}^{I}$ can be defined as

(A2) $$OP_{U_{1}}^{I}=1-\left[\int_{0}^{\frac{\rho_{I}}{{\bar{\rho }}_{S}}}f_{{\left|h_{SP}\right|}^{2}}\left(x\right)\int_{\frac{\theta }{{\bar{\rho }}_{S}}}^{\infty }f_{{\left|h_{1}\right|}^{2}}\left(y\right)dydx+\int_{0}^{\frac{\rho_{I}}{{\bar{\rho }}_{S}}}f_{{\left|h_{SP}\right|}^{2}}\left(x\right)\int_{\frac{\theta }{{\bar{\rho }}_{S}}}^{\infty }f_{{\left|h_{1}\right|}^{2}}\left(y\right)dydx\right]$$

where $\rho_{I}=\frac{I_{th}}{N_{0}}$ stands for the temperature-constraint-to-noise ratio at PD. Thus, with the help of Equations (5) and (6), we can calculate as

(A3) $$OP_{U_{1}}^{I}=1-\left[e^{-\frac{\theta }{{\bar{\rho }}_{S}\lambda_{1}}}\left(1-e^{-\frac{\rho_{I}}{{\bar{\rho }}_{S}\lambda_{SP}}}\right)+\ell_{1}e^{-\left(\frac{\rho_{I}\lambda_{1}+\lambda_{SP}\theta }{{\bar{\rho }}_{S}\lambda_{1}\lambda_{SP}}\right)}\right]$$

where $\theta =\frac{\upsilon }{\left(a_{1}-\varepsilon_{1}a_{2}\right)}$, $\upsilon =\varepsilon_{1}\left(\xi +1\right)$ and $\ell_{1}=\frac{\lambda_{1}\rho_{I}}{\lambda_{SP}\theta +\lambda_{1}\rho_{I}}$. This is the end of the proof. □

## Appendix B

**Proof** **of** **Lemma** **1.** By invoking Equations (2) and (3) into Equation (19), we can express $A_{1}$ as

(A4) $$\begin{array}{c} A_{1}=\mathrm{Pr}\left(\frac{{\bar{\rho }}_{S}a_{1}{\left|h_{1}\right|}^{2}}{{\bar{\rho }}_{S}a_{2}{\left|h_{1}\right|}^{2}+\left(\xi +1\right)}<\varepsilon_{1},\frac{{\bar{\rho }}_{S}a_{1}{\left|h_{2}\right|}^{2}}{{\bar{\rho }}_{S}a_{2}{\left|h_{2}\right|}^{2}+\left(\xi +1\right)}<\varepsilon_{1}\right.\left.,{\bar{\rho }}_{S}<\frac{\rho_{I}}{{\left|h_{SP}\right|}^{2}}\right)\\ +\mathrm{Pr}\left(\frac{\rho_{I}a_{1}{\left|h_{1}\right|}^{2}}{\rho_{I}a_{2}{\left|h_{1}\right|}^{2}+{\left|h_{SP}\right|}^{2}\left(\xi +1\right)}<\varepsilon_{1}\right.\left.,\frac{\rho_{I}a_{1}{\left|h_{2}\right|}^{2}}{\rho_{I}a_{2}{\left|h_{2}\right|}^{2}+{\left|h_{SP}\right|}^{2}\left(\xi +1\right)}<\varepsilon_{1},{\bar{\rho }}_{S}<\frac{\rho_{I}}{{\left|h_{SP}\right|}^{2}}\right) \end{array}$$

First, we define the first and second term of Equation (A4) as $A_{1,1}$ and $A_{1,2}$, respectively. In particular, $A_{1,1}$ is defined as

(A5) $$A_{1,1}=\mathrm{Pr}\left({\left|h_{1}\right|}^{2}<\frac{\theta }{{\bar{\rho }}_{S}},{\left|h_{2}\right|}^{2}<\frac{\theta }{{\bar{\rho }}_{S}},{\left|h_{SP}\right|}^{2}<\frac{\rho_{I}}{{\bar{\rho }}_{S}}\right)$$

We have

(A6) $$A_{1,1}=\left(1-e^{\frac{\theta }{\lambda_{1}{\bar{\rho }}_{S}}}\right)\left(1-e^{\frac{\theta }{\lambda_{2}{\bar{\rho }}_{S}}}\right)\left(1-e^{-\frac{\rho_{I}}{\lambda_{SP}{\bar{\rho }}_{S}}}\right)$$

Thus, the term $A_{1,2}$ can be further formulated as

(A7) $$\begin{array}{c} A_{1,2}=\mathrm{Pr}\left({\left|h_{1}\right|}^{2}<\frac{\theta {\left|h_{SP}\right|}^{2}}{\rho_{I}},{\left|h_{2}\right|}^{2}<\frac{\theta {\left|h_{SP}\right|}^{2}}{\rho_{I}}\right.\left.,{\left|h_{SP}\right|}^{2}>\frac{\rho_{I}}{{\bar{\rho }}_{S}}\right) \end{array}$$

Next, it can be calculated as

(A8) $$A_{1,2}=\int_{\frac{\rho_{I}}{{\bar{\rho }}_{S}}}^{\infty }f_{{\left|h_{SP}\right|}^{2}}\left(x\right)\int_{0}^{\frac{\theta x}{\rho_{I}}}f_{{\left|h_{1}\right|}^{2}}\left(y\right)\int_{0}^{\frac{\theta x}{\rho_{I}}}f_{{\left|h_{1}\right|}^{2}}\left(z\right)dzdydx$$

Finally, $A_{1,2}$ is written as

(A9) $$\begin{array}{c} A_{1,2}=\left[e^{-\frac{\rho_{I}}{{\bar{\rho }}_{S}\lambda_{SP}}}-\ell_{1}e^{-\frac{\lambda_{SP}\theta +\lambda_{1}\rho_{I}}{\lambda_{SP}\lambda_{1}{\bar{\rho }}_{S}}}\right.\left.-\ell_{2}e^{-\frac{\lambda_{SP}\theta +\lambda_{2}\rho_{I}}{\lambda_{SP}\lambda_{2}{\bar{\rho }}_{S}}}+\ell_{3}e^{-\frac{\lambda_{SP}\lambda_{2}\theta +\lambda_{SP}\lambda_{1}\theta +\lambda_{1}\lambda_{2}\rho_{I}}{\lambda_{SP}\lambda_{1}\lambda_{2}{\bar{\rho }}_{S}}}\right] \end{array}$$

where $\ell_{2}=\frac{\lambda_{2}\rho_{I}}{\lambda_{SP}\theta +\lambda_{2}\rho_{I}}$ and $\ell_{3}=\frac{\lambda_{1}\lambda_{2}\rho_{I}}{\lambda_{SP}\lambda_{2}\theta +\lambda_{SP}\lambda_{1}\theta +\lambda_{1}\lambda_{2}\rho_{I}}$. This is the end of the proof. □
